# Revisiting the role of education in attitudes toward immigration in different contexts in Europe

**DOI:** 10.1186/s41118-024-00238-9

**Published:** 2025-01-03

**Authors:** Karen Umansky, Daniela Weber, Wolfgang Lutz

**Affiliations:** 1 Faculty of Economics and Social Sciences, University of Potsdam, 14482 Potsdam, Germany; 2 Health Economics and Policy Division, Vienna University of Economics and Business, 1020 Vienna, Austria; 3 International Institute for Applied Systems Analysis (IIASA), Wittgenstein Centre for Demography and Global Human Capital (IIASA, OeAW, University of Vienna), 2361 Laxenburg, Austria; 4 Department of Demography, University of Vienna, Wittgenstein Centre for Demography and Global Human Capital (IIASA, OeAW, University of Vienna), 1010 Vienna, Austria; 5 Vienna Institute of Demography, Austrian Academy of Sciences, Wittgenstein Centre for Demography and Global Human Capital (IIASA, OeAW, University of Vienna), 1010 Vienna, Austria

**Keywords:** Immigration, Attitudes toward immigration, Education, European Social Survey, Europe

## Abstract

Among the individual determinants of attitudes toward immigration, the liberalising role of education is well known—those with higher levels of education tend to be more in favour of immigration. However, recent socioeconomic changes and idiosyncratic differences between European countries prompt us to reassess the role of education, given these contextual differences. Does it still apply, and is it universal? Moreover, does this relationship apply to both cultural and economic attitudes toward immigration? Using data from the European Social Survey, we analyse the role of education and socioeconomic changes in shaping economic and cultural attitudes toward immigration in 15 European countries over 16 years using a hierarchical model with cross-classified random effects. In our analysis, we distinguish between Eastern European and non-Eastern European countries. Our results indicate a robust positive and significant association between higher levels of education and more tolerant attitudes toward immigration in both aspects. However, they also reveal that the strength of this relationship varies between the two attitudes by context and region. For example, higher migrant inflow rates attenuate education’s liberalising and empowering role in shaping cultural attitudes in non-Eastern European countries but are not significant in Eastern European countries. Thus, our findings contribute to the literature examining the role of context in the established relationship between education and immigration attitudes while providing insights into regional differences.

**Supplementary Information:**

The online version contains supplementary material available at 10.1186/s41118-024-00238-9.

## Introduction

The American writer and political activist Helen Keller once said that “the highest result of education is tolerance” (Keller, 1903: 44). Higher education is generally seen as a panacea for anti-immigration attitudes. Among the individual predictors of attitudes toward immigration, its positive association with liberal, pro-immigration attitudes and negative association with ethnic exclusion and national chauvinism has been so widely documented (Borgonovi & Pokropek, 2019; Ceobanu & Escandell, 2010; Coenders & Scheepers, 2003; Dražanová et al., 2023; Hainmueller & Hiscox, 2007) that some even concede a so-called “liberalising effect”. The actual causality of the relationship is notoriously challenging to establish, and studies that attempt this endeavour come to no definitive conclusion. Individual country studies either find no evidence for the causal link (Finseraas et al., 2018; Weber, 2022) or only a small direct causal effect on individuals’ attitudes, which is not always liberalising (Simon, 2022), or a small but liberalising effect (Velásquez & Eger, 2022). Conversely, a study of five Western European countries demonstrates that an additional year of secondary education substantially decreases anti-immigration attitudes (Cavaille & Marshall, 2019). These inconsistencies reveal that the role of context may be as important as differences between levels of educational attainment. If so, could these inconsistencies be related to recent changes in the social and economic environment?

The first two decades of the third millennium were a challenging time for all European countries—the Great Recession of 2008, followed by the Eurozone crisis and the 2015 refugee crisis, altered not only the living conditions but also the very fabric of European societies. In addition to the existing idiosyncratic differences between European countries, these socioeconomic changes impacted them asymmetrically, affecting some more or differently than others. In times like these, the role of context comes to the fore—how do socioeconomic changes and regional differences affect attitudes toward immigration?

Group threat theory proponents emphasise cultural and economic threats—real or perceived—as possible determinants of immigration attitudes (Lubbers & Scheepers, 2007; Stephan & Stephan, 1996). Group members may become hostile toward other groups if they feel that their national or economic interests are threatened (Jackson et al., 2001; Scheepers et al., 2002). Indeed, recent literature investigating the impact of economic downturns reports that attitudes toward immigration became less favourable during the great recession, particularly in countries severely affected by it (Hatton, 2014; Isaksen, 2019). Studies analysing the possible impact of the refugee crisis on attitudes toward immigration come to a less universal conclusion. Some found a polarising effect for Western Europe but no significant effect for South and Central Eastern Europe (van der Brug & Harteveld, 2021), while others found no evidence that the refugee crisis increased anti-immigration attitudes in Europe (Stockemer et al., 2020).

More important for our undertaking is to understand how these socioeconomic changes affect established relationships, such as that between education and immigration attitudes. Studies suggest that less educated individuals from lower socioeconomic strata are more susceptible to perceptions of cultural and economic threat (Konings & Mosaico, 2020; Manevska & Achterberg, 2013). Similarly, higher-educated individuals should be more immune to socioeconomic changes and less susceptible to perceived cultural or economic threats from immigrants. Velásquez and Eger (2022), who examined the impact of the refugee crisis on the association between education and immigration attitudes in Norway, found that education has such an inoculating effect in the face of the crisis.

The literature on attitudes toward immigration emphasises the need for a broader range of studies researching contextual effects (Dinesen & Hjorth, 2020; Dražanová, 2022). Few recent studies critically examined the role of education in shaping immigration attitudes in different contextual settings over time. Borgonovi and Pokropek (2019), who establish the importance of contextual factors to the strength of this relationship, and Eick (2024), who finds welfare chauvinism even in higher educated socioeconomic strata, are among the notable exceptions. However, they do not consider cultural and economic attitudes separately, possibly reflecting the two corresponding types of perceived threats. Moreover, it appears that scholarly attention has primarily focused on analysing attitudes toward immigration in Western European countries, while research on non-Western European countries is scarce (Dinesen & Hjorth, 2020) and finds little evidence for the relationship in the non-Western area (Dražanová, 2017).

Our study aims to better understand if the relationship between higher education and more inclusive attitudes toward immigration still holds, given the broader context of recent socioeconomic changes and idiosyncratic differences among European countries. Our central argument is that education’s liberalising role is present but may vary under different contextual circumstances. More specifically, we expect variation in different aspects of attitudes toward immigration, namely, some contextual factors may come into play when defining the role of education in shaping cultural attitudes, while others may trigger changes in economic attitudes. We, thus, bring together individual and contextual factors to explain differences in attitudes toward immigration across countries and over time, with particular attention to regional differences.

Our contribution begins by analysing two aspects of immigration attitudes separately rather than combining them into a single index. Culturally, natives may view immigration as enriching or undermining their society, while economically, they may view immigration as improving or worsening the country’s economy. Interestingly, no correlation is warranted between the two aspects. Natives may welcome immigration, because it brings economic benefits but may oppose it, because they reject cultural diversity and vice versa. Thus, we distinguish these two aspects and identify possible attitudinal differences between subpopulations with different educational backgrounds. In addition, we examine the role of education in shaping cultural and economic aspects of attitudes toward immigration and its interaction with contextual variables. To this end, we consider country-specific period effects, such as changes in the inflows of foreign-born populations and unemployment rates. Using data from the European Social Survey, we study 15 European countries—12 non-Eastern European and 3 Eastern European—between 2002 and 2018.

Our findings lend support to the liberalising and empowering role of education, with higher-educated individuals being more open to immigration and less threatened by it in both aspects than their less-educated peers. We find, however, that context matters and that some socioeconomic changes tend to mitigate this effect. By discussing the role of contextual and regional factors in this relationship, we aim to promote a broader and deeper understanding of the role of higher education in defining cultural and economic attitudes toward immigration.

## Theoretical background

Scholarly interest in the question of what determines individual perceptions of immigration prompted theories at individual and contextual levels. The former focus on demographics, such as level of education (Ceobanu & Escandell, 2008, 2010; Coenders & Scheepers, 2003; Dražanová et al., 2023; Hainmueller & Hiscox, 2007), age and cohort (Coenders et al., 2008; Dražanová et al., 2023; Gorodzeisky & Semyonov, 2018; Jeannet & Dražanová, 2023), as well as gender (Ceobanu & Escandell, 2008; Gorodzeisky & Semyonov, 2009; Ponce, 2017; Semyonov et al., 2006). In addition, they identify political affiliation (Chandler & Tsai, 2001; Citrin et al., 1997; Harteveld et al., 2017; Mayda, 2006; Sides & Citrin, 2007), place of residence (Dražanová et al., 2023; Gorodzeisky & Semyonov, 2009; McLaren, 2003), whether an individual was born in the country they live in (Braakmann et al., 2017; Gorodzeisky & Semyonov, 2009), and individual satisfaction with the economy (Miller, 2012). Moreover, scholarship emphasises the impact of contextual factors such as changes in the economic situation (Dancygier & Donnelly, 2014; Hatton, 2014; Isaksen, 2019) and the cultural composition of societies (Hopkins, 2010; van der Brug & Harteveld, 2021), although the literature on contextual factors is somewhat limited (Dražanová, 2022).

Among the individual-level variables, scholarship considers education to be one of the most stable predictors of attitudes toward immigration—higher-educated individuals tend to be more inclusive toward foreign groups and welcome immigration (Dražanová et al., 2023). More than only a proxy for socioeconomic status, it is assumed to have a liberalising effect on immigration attitudes (e.g., Velásquez & Eger, 2022). Furthermore, it is assumed that the mechanism by which educational attainment influences attitudes toward migration is a mixture of enhanced cognitive ability associated with better powers of abstraction and broader intellectual horizons, liberal societal norms and values endorsed by higher educational institutions, and better socioeconomic status, which makes immigration appear less threatening in terms of competition (Ceobanu & Escandel, 2008; Dražanová et al., 2023; Gorodzeisky & Semyonov, 2009; Hyman & Wright, 1979; Weil, 1985). Some recent studies, however, report welfare chauvinistic attitudes toward immigration, even among highly educated Europeans (Eick, 2024).

Our central argument is that variance in this well-established relationship can be attributed to contextual factors such as socioeconomic changes or regional differences. For example, if immigration attitudes decline during economic downturns (Dancygier & Donnelly, 2014; Hatton, 2014; Isaksen, 2019) or rapid changes in population composition (Hopkins, 2010), or if there are regional discrepancies between Eastern European and non-Eastern European countries (Bell et al., 2021), it is plausible to expect these contextual factors to impact education’s liberalising role in defining attitudes toward immigration.

Many approaches have in common that the perception of a threat posed by immigration to natives individually or collectively affects immigration attitudes (Ceobanu & Escandell, 2010). Group threat theory—the predominant theory explaining attitudes toward immigration—assumes that individuals belonging to a particular group (in-group) view it more favourably than other groups (out-groups) (Mummendey et al., 2001; Sniderman et al., 2004; Tajfel & Turner, 1986). This inevitably implies hostility toward out-groups, as they challenge the homogeneity of the in-group. More specifically, in-group members may become hostile toward out-groups when they feel that their national or economic interests are threatened (Jackson et al., 2001; Scheepers et al., 2002).

A perceived cultural threat is based on the fear that the identity of the out-group could jeopardise the distinct national identity, worldview, values, and traditions of the in-group (Lubbers & Scheepers, 2007; Sniderman et al., 2004; Stephan & Stephan, 1996). Previous research found strong evidence for the role of cultural threat in defining immigration attitudes (Malhotra et al., 2013). Moreover, it was reported that higher-educated individuals are more likely to favour cultural diversity (Hainmueller & Hiscox, 2007), as higher education is often associated with support for liberal values (Hyman & Wright, 1979).

A perceived economic threat can manifest in egotropic and sociotropic guises. Egotropic concerns are often related to the labour–market competition hypothesis, whereby native lower socioeconomic strata may fear competition with immigrants for available lower-status jobs, leading to unfavourable attitudes toward immigration (Scheve & Slaughter, 2001; Sides & Citrin, 2007). Times of economic hardships appear to exacerbate this correlation (Burns & Gimpel, 2000; Semyonov et al., 2008). Conversely, studies found that a strong position in the labour market and a higher occupational classification, which is often tied to higher education, are directly linked to a positive attitude toward immigration (Dražanová et al., 2023; Gorodzeisky & Semyonov, 2009). However, the premise of the labour–market competition—that natives would oppose immigrants with a similar socioeconomic level and skill set as themselves—was refuted by other studies (Dinesen et al., 2016; Hainmueller & Hiscox, 2007). They found that, regardless of their socioeconomic status, natives favoured better-educated, highly skilled immigrants (Dinesen & Hjorth, 2020).

From a sociotropic perspective, negative views of the national economy are associated with less favourable immigration attitudes (Citrin et al., 1997; Sides & Citrin, 2007). Natives may fear that immigrants put the national welfare system under strain, increasing competition for the distribution of welfare resources (Hainmueller & Hiscox, 2010). Furthermore, the cost of accommodating immigrants may be reflected in the taxes paid by higher earners, resulting in anti-immigration sentiments in the higher socioeconomic strata (Hainmueller & Hopkins, 2014). The literature, therefore, agrees that opposition to immigration is primarily driven by national sociotropic rather than egotropic concerns (Dinesen & Hjorth, 2020; Dražanová et al., 2023).

The recent socioeconomic changes in Europe make the aggravation of the perceived cultural and economic threats plausible. The economic crises of the first two decades of the third millennium put the remnants of national welfare systems in Europe under pressure; economic security and solidarity gave way to austerity. According to the hypothesis that economic hardship breeds extremism, we expect it to have influenced the perception of the economic threat out-groups pose. The same could be true, given the ethnocultural diversity fostered by the recent influx of immigrants and refugees into European countries. These changes, however, did not affect all European countries equally. The Eurozone crisis, for example, hit the so-called “PIIGS” group of countries comprising Portugal, Ireland, Italy, Greece, and Spain the hardest. These countries not only suffered from a deep economic recession but also had to implement harsh austerity measures imposed by the EU (Duman, 2017). The impact of the 2015 refugee crisis was also asymmetrical among European countries. Germany, Norway, Sweden, and Switzerland were popular destination countries that received the most asylum seekers. In contrast, countries such as Greece, Italy, Hungary, and Slovenia were transit countries due to their location on the European entry routes, with Hungary recording the highest number of asylum seekers (Pew Research Center, 2016).

These socioeconomic changes require a reassessment of the established predictors of attitudes toward immigration. Will the liberalising and empowering role of higher education in shaping economic and cultural attitudes toward immigration change significantly in the face of these changes? We conceptualise the liberalising role of education in shaping cultural and economic attitudes as the difference in attitudes between higher-educated and lower-educated people. Our study tests the hypothesis that higher education—a mix of enhanced cognitive skills associated with better abstracting ability, broader intellectual horizons, greater openness to cultural diversity, and better socioeconomic status—is empowering and thus makes immigration less threatening in terms of economic and cultural competition, considering regional differences and changing rates of unemployment and influx of immigrants over the years:

- H1: The liberalising role of education remains positive and significant in both aspects, even in times of a high influx of immigrants and economic hardship.

Following our theoretical premise, we expect some contextual factors to be more prominent in defining the role of education in shaping cultural attitudes and others in shaping economic attitudes. This further supports our decision to examine these two dimensions of immigration attitudes separately rather than combining them into a single index (e.g., McLaren & Paterson, 2020). For example, fluctuations in migration rates, such as during the European refugee crisis, may catalyse negative cultural attitudes toward immigration, thereby affecting education’s liberalising role. Group conflict theory, particularly integrated threat theory, suggests that a high influx of migrants may increase perceptions of competition over intangible cultural resources, such as in-group values, tradition, identity, and language (Stephan & Stephan, 1996). This perceived cultural threat will likely foster unfavourable attitudes toward immigration (Scheepers et al., 2002). As higher education is generally associated with more liberal values (Hyman & Wrights, 1979), higher educated are more supportive of cultural diversity (Hainmueller & Hiscox, 2007) and less prone to prejudice (Coenders & Scheepers, 2003) than lower educated. We, therefore, expect the gap in cultural attitudes toward immigration between higher and lower educated to widen as the influx of migrants increases and cultural diversity becomes more apparent.

A high influx of immigrants can also heighten perceptions of competition over tangible resources, such as access to social benefits and tax burdens, thus intensifying perceptions of an economic threat. Previous studies suggest that less educated individuals from lower socioeconomic backgrounds are particularly susceptible to threat perceptions (Konings & Mosaico, 2020; Manevska & Achterberg, 2013). Other studies indicate that higher socioeconomic strata may also develop less favourable immigration attitudes, because they fear the costs of accommodating immigrants through higher taxes (Hainmueller & Hopkins, 2014). Therefore, we do not expect the difference in economic attitudes between higher and lower educated to increase significantly in times of increased migration inflows as both lower and higher educated may perceive it as an economic threat:

- H2: A higher influx of immigrants will increase education’s liberalising role for cultural attitudes but not for economic ones.

Rising unemployment rates, such as during the Great Recession and the European sovereign debt crisis, are associated with increasingly hostile attitudes toward immigration (Hatton, 2014; Isaksen, 2019) and can be expected to affect the liberalising role of education. If immigrants are assumed to be less educated and compete for similar types of jobs as the less educated natives belonging to the lower socioeconomic strata, we may expect the latter to develop negative attitudes toward immigration due to perceived competition for lower-status jobs (Scheve & Slaughter, 2001; Sides & Citrin, 2007). In contrast, better-educated individuals in higher socioeconomic strata, often associated with more favourable immigration attitudes (Dražanová et al., 2023; Gorodzeisky & Semyonov, 2009), are typically employed in professions less affected by immigration, which reduces the likelihood of direct job competition. Therefore, we expect that economic hardship, proxied by fluctuations in unemployment rates, will widen the gap in economic attitudes toward immigration between the lower and higher educated.

Economic factors such as rising unemployment appear to influence not only economic but also cultural attitudes toward immigration (Hainmueller & Hopkins, 2014). Studies documented a so-called “spillover effect”, whereby economic hardship amplifies cultural concerns about immigration, accentuating in-group versus out-group distinctions, particularly among economically vulnerable, lower-educated individuals (e.g., Semyonov et al., 2006). Conversely, higher-educated individuals, who are typically more open to cultural diversity, are less likely to perceive cultural threats in times of economic downturn due to a relatively stable socioeconomic status and more inclusive value systems (Hainmueller & Hopkins, 2014). Therefore, we expect the “spillover effect” of economic hardship on cultural attitudes toward immigration mostly among lower-educated individuals, reinforcing education’s liberalising role:

- H3: Economic hardship will increase education’s liberalising role for both economic and cultural attitudes.

In addition, this study examines regional differences between Eastern European and non-Eastern European countries to contribute to the scarce literature studying immigration attitudes in non-Anglo contexts (e.g., Bell et al., 2021; Dražanová, 2017).

## Data and methods

### Data

In this study, we analyse data from the European Social Survey (ESS)—a cross-national survey that began in 2002 and is conducted every 2 years with face-to-face interviews. The data set offers two separate questions on cultural and economic aspects of attitudes toward immigration, which have the same wording in all nine rounds and are relevant to this study. We use data from 15 countries (Belgium, Finland, France, Germany, Hungary, Ireland, the Netherlands, Norway, Poland, Portugal, Slovenia, Spain, Sweden, Switzerland, and the United Kingdom) that participated in all nine waves. Furthermore, we use data from FAOSTAT (2020) for population size, OECD (2020) and SiStat (2020) for migrant inflows, and data from the World Bank for unemployment rates (World Bank, 2023).

Our two outcome variables—cultural and economic attitudes toward immigration—were addressed by the following questions, with responses rated on an 11-point Likert scale:

- 1. A country’s cultural life is undermined or enriched by immigrants.
- Would you say that [respondent’s country]’s cultural life is generally undermined or enriched by people coming to live here from other countries? (0 = cultural life undermined; 10 = cultural life enriched).
- 2. Immigration is bad or good for a country’s economy.
- Would you say it is generally bad or good for [respondent’s country]’s economy that people come to live here from other countries? (0 = bad for the economy; 10 = good for the economy).

Participants’ individual level of education was classified into five ISCED categories using the International Standard Classification of Education (ISCED-97): (1) less than lower secondary education (ISCED I); (2) lower secondary education (ISCED II); (3) upper secondary education (ISCED III); (4) advanced vocational education (ISCED IV); and (5) at least lower tertiary education, B.A. level (ISCED V).

Among the individual control variables, we include satisfaction with the economy (0 = extremely dissatisfied; 10 = extremely satisfied), following the literature that shows that natives have unfavourable attitudes toward immigration in times of economic hardship (e.g., Burns & Gimpel, 2000). In addition, we consider gender and whether a respondent is from the country they live in, as these were shown to affect attitudes toward immigration (Gorodzeisky & Semyonov, 2009). Moreover, we consider the contact theory, according to which a higher proportion of immigrants are found in big cities than in rural areas (Bell et al., 2010), by including a variable for residence (0 = big cities; 1 = rural areas). We also account for left–right political affiliation (0 = left; 10 = right) as exposure to right-wing populist rhetoric was found to promote anti-immigrant attitudes (Harteveld et al., 2017; Heiss & Matthes, 2020). The age of the participants, ranging from 14 to 123 years, was categorised into 10-year groups, as some studies found a non-linear association between age and attitudes toward immigration (e.g., Coenders & Scheepers, 2003).

We consider the following contextual factors in our analysis: (1) the migrant inflow rate, i.e., the foreign population that immigrated to a country in the year prior to the respective survey year, divided by the country’s total population and presented in percentage points (summary statistics are provided in Table A1 in the appendix); (2) the unemployment rate in the survey year, which indicates the share of the labour force that was unemployed but was actively seeking employment. These contextual factors represent a changing socioeconomic environment. In addition, they may account for a possible cultural and economic threat from newcomers, as natives can develop hostile attitudes toward immigration if their cultural and economic interests are threatened (Jackson et al., 2001; Scheepers et al., 2002).

### Methods

We investigate the role of education and contextual factors (i.e., migrant inflow rate and unemployment rate) in shaping the two aspects of respondents’ attitudes toward immigration (cultural and economic) by examining the sample from 15 European countries that participated in nine ESS waves over 16 years. We begin our analysis with a descriptive overview of net attitudes toward immigration in cultural and economic aspects in the selected countries. For the descriptive statistics, we convert the 11-point Likert scale of both variables on attitudes toward immigration into a binary positive/negative attitude, using five as the cutoff point: All values greater than five were coded as positive, while values equal to or less than five were coded as negative attitudes toward immigration. To estimate the net percentage, we subtracted the percentage of respondents who reported negative attitudes toward immigration from those who had positive attitudes in each aspect for each country. We report average cultural and economic attitudes toward immigration for non-Eastern European and Eastern European countries. In addition, we present net attitudes for each survey year over 16 years for non-Eastern and Eastern European countries as well as the entire sample.

There is evidence in the literature of temporal trends in attitudes toward immigration, which could be period or cohort effects. For example, McLaren and Paterson (2020) identified cohort effects in European data, Wilkes and Corrigall-Brown (2011) found a strong period effect and only a small cohort effect in Canadian data, while Beller (2020) found cohort and period effects when analysing German data. However, the linearity between age, period, and cohorts requires special consideration in the modelling strategy. Therefore, following the statistics and migration literature, we address the potential confounding influence of age and periods by applying hierarchical age-period-cohort regression analysis (HAPC) (Jeannet & Dražanová, 2023; McLaren & Paterson, 2020; Yang & Land, 2013). More specifically, we apply a hierarchical cross-classified random effects model (Beller, 2020; Jeannet & Dražanová, 2023; McLaren & Paterson, 2020; Wilkes & Corrigall-Brown, 2011; Yang & Land, 2006). This model allows us to account for the hierarchical structure of the unbalanced repeated cross-sectional data, with individuals being cross-classified and nested in both country-period and country-cohort while clustered in 15 European countries. Thus, we estimate the variability across periods and cohorts while testing the role of individual education and contextual (country-period) factors. In our approach, we enter age as a level one variable, country-period and country-cohort as cross-classified level two units, and country as a level three variable. Moreover, we add individual-level variables to the participants’ characteristics, such as education, gender, place of residence, native population group membership, left–right party affiliation, satisfaction with the economy and country-period variables, and contextual variables, such as migrant inflow rate and unemployment rate.

The two aspects of attitudes toward immigration are analysed separately with individual-level (Level 1), country-cohort (Level 2), country-year (Level 2), and country-level (Level 3) effects. We ran the following models for each outcome variable: First, we estimate a null hierarchical three-level cross-classified model (Model 0). Model 1 examines the effect of education on cultural and economic attitudes toward immigration while controlling for individual-level factors. Then, we add contextual country-period factors on socioeconomic changes with model 2a, including the migrant inflow rate and model 2b, including the unemployment rate. In model 3a, the interaction between individual education and country-period variable migrant inflow rate is added, whereas model 3b adds the interaction between individual education and the unemployment rate. Model 3 can be written as

$${y}_{ijkl}={\upbeta }_{0}+{\upbeta }_{1}\,{X}_{ijkl}+{\upbeta }_{2}\,Edu{c}_{ijkl}+{\upbeta }_{3}{T}_{kl}+{\upbeta }_{4}Edu{c}_{ijkl}\times {T}_{kl}+{u}_{0jl}+{\upnu }_{0kl}+{\upomega }_{0l}+{e}_{0ijkl}$$

where $y_{ijkl}$ is the attitude toward migration score for individual i within country-cohort j, country-period k, and country l. Furthermore, ${X}_{ijkl}$ is a vector of the individual-level factors we control for, $Edu{c}_{ijkl}$ is the individual education, ${T}_{kl}$ is the country-period characteristics (either the migrant inflow rate or the unemployment rate), and the residual random effects of individual (${e}_{0ijkl}$), country-cohort (${u}_{0jl}$), country-period (${\upnu }_{0kl}$), and country (${\upomega }_{0l}$), where the random effects are all assumed to be normally distributed with mean 0.

Moreover, we consider regional differences in our country pool, namely, the division into Eastern and non-Eastern Europe. Therefore, we ran all our models separately on the subsample of Eastern European countries (Hungary, Poland, and Slovenia) and Non-Eastern European countries. In our analysis, we corrected for non-response and population size by including survey weights.

## Results

### Descriptive results

First, we examine how attitudes toward immigration differ culturally and economically across the European countries in our sample. Table 1 reports individual net attitudes toward immigration in cultural and economic aspects in the 15 selected countries, with the top and bottom 15% of values highlighted in bold to facilitate comparison. On average, respondents tended to have positive attitudes toward immigration in cultural terms (14.6%) but negative attitudes in economic terms (− 12.8%) (Table 1).

**Table 1:** Net share of respondents reporting cultural and economic attitudes toward immigration by country

Country	Net cultural attitude	Net economic attitude
*Non-Eastern Europe*		
Belgium	13.9	− 29.2
Finland	**59.3**	− 4.0
France	− 6.9	− 28.6
Germany	14.3	− 6.5
Ireland	10.5	− 0.9
Netherlands	33.1	− 11.7
Norway	10.9	− 0.7
Portugal	2.1	− 14.4
Spain	22.5	− 1.6
Sweden	**51.5**	**3.6**
Switzerland	21.6	**18.7**
United Kingdom	− 5.5	− 19.5
**Average**	18.9	− 7.9
**Eastern Europe**		
Hungary	− **17.4**	− **50.5**
Poland	27.0	− 6.5
Slovenia	−** 18.6**	−** 39.4**
**Average**	− 3.0	− 32.2
**Total Sample Average**	14.6	− 12.8

Values on the 11-point Likert scale above five were summarised as positive attitudes, while values less than or equal to five were coded as negative attitudes; the net percentage was calculated by subtracting the percentage of respondents indicating a negative attitude from those indicating a positive attitude in each aspect; the top and bottom 15% values are in bold; survey weights were considered

Source: European Social Survey 2002–2018, 15 European countries

The non-Eastern European sub-sample supports this trend, demonstrating even greater tolerance toward immigration in the cultural aspect (18.9%) and less intolerance in the economic aspect (− 7.9%) than the general sample. This tendency was consistent with most of the countries in the subsample, except for Sweden and Switzerland—the only two countries with positive attitudes toward immigration also in the economic aspect (3.6% and 18.7%, respectively)—and France and the UK, which showed negative attitudes in both aspects to varying degrees. The most positive attitudes were found in Finland and Sweden, which appear to be the most culturally favourable toward immigration, with 59.3% and 51.5% of respondents, respectively.

The Eastern European sub-sample, however, indicates that, on average, respondents had predominantly negative attitudes toward immigration in both aspects. Specifically, intolerance toward immigration was lower in the cultural aspect (− 3%) than in the economic aspect (− 32.2%). Hungary and Slovenia showed the most negative net attitudes toward immigration in both aspects, with Slovenia (− 18.6%) in the cultural aspect and Hungary (− 50.5%) in the economic aspect, achieving the lowest score not only in the Eastern European sub-sample but also among all 15 countries. In contrast, the attitudes reported by Poland were more in line with those of the non-Eastern European sub-sample, showing positive attitudes in the cultural aspect (27%) and slightly negative attitudes in the economic aspect (− 6.5%).

The division of the sample into Eastern European and non-Eastern European countries revealed that, on average, the three Eastern European countries had a negative net attitude in cultural and especially economic terms, while the average net attitudes of the non-Eastern European countries were above the average of the total sample. Despite the idiosyncratic differences in attitudes toward immigration, the descriptive results at the individual country level confirm the general trend that Europeans are less supportive of immigration from an economic perspective than from a cultural perspective. According to these findings, developing an economic threat perception is more likely than a cultural one.

**Fig. 1:**
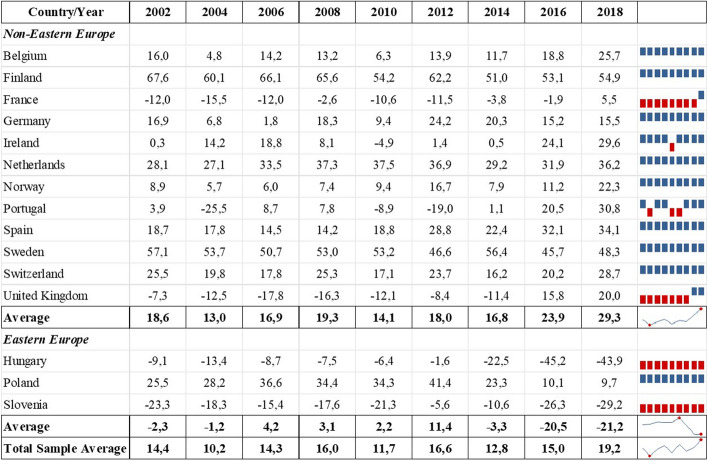
Net share of respondents reporting cultural attitudes toward immigration by country and year. Source: European Social Survey, 2002–2018. The net percentage was calculated by subtracting the percentage of respondents who indicated a negative cultural attitude from those with a positive cultural attitude; sampling weights were considered

**Fig. 2:**
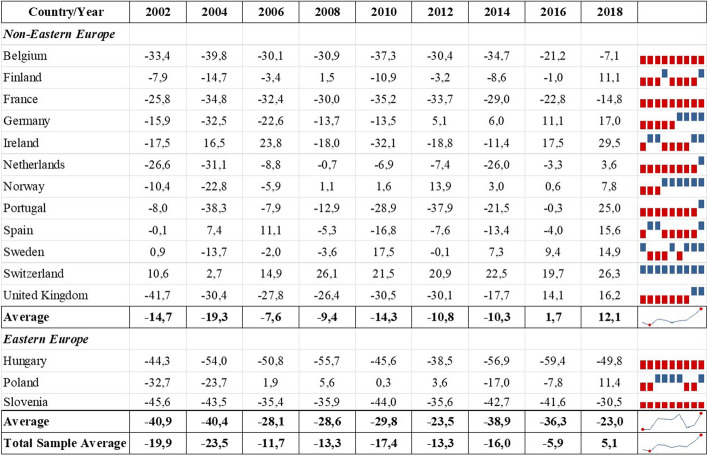
Net share of respondents reporting economic attitudes toward immigration by country and year. Source: European Social Survey, 2002–2018. The net percentage was calculated by subtracting the percentage of respondents who indicated a negative economic attitude from those with a positive economic attitude; sampling weights were considered

Using average scores over such a long period could hide important temporal effects. Therefore, we examine how cultural and economic attitudes toward immigration vary over time in our sample. Figures 1 and 2 show the evolution of net cultural and economic attitudes toward immigration in non-Eastern and Eastern European countries over 16 years and the average net attitudes per year, respectively. The sparklines on the right side of the figures mark the positive net attitudes of the countries in blue and the negative ones in red. The mini graphs at the bottom of the sparklines show the evolution of cultural or economic attitudes in each sub-sample and the total sample of countries over time.

Following the group threat theory literature, we expected the Eurozone crisis and the European refugee crisis to affect attitudes toward immigration negatively. The average values for the entire sample indicate that the net attitude toward immigration in cultural and economic aspects dropped in 2010, but this was reversed in 2012. Thus, the Eurozone crisis may have been associated with a short-term decline in attitudes toward immigration. In contrast, average net attitudes toward immigration in both aspects increased steadily between 2014 and 2018, indicating a more welcoming stance across the entire sample. Overall, our descriptive results indicate that the refugee crisis was not linked to less favourable net attitudes toward immigration—quite the opposite. This corroborates previous findings (Dennison & Geddes, 2019) and refutes our expectation that the refugee crisis might have reduced positive attitudes toward immigration.

Interestingly, the least positive total average net attitude toward immigration in both aspects was in 2004. While the number of refugees worldwide declined sharply in 2003 (UNHCR, 2004), European countries faced the most extensive enlargement of the EU when ten new Member States (seven of them from Eastern Europe, including Hungary, Poland, and Slovenia) joined the EU in May 2004. This considerable wave of EU enlargement may have raised doubts among existing Member States about how it might affect their cultural and economic situation. Separating the Eastern European countries from the rest of our sample helps to clarify this point, as the negative slump in 2004 occurred only among the non-Eastern European countries (Figs. 1 and 2). It also urges a closer look at the differences between our sub-samples.

The mini graphs for the sub-samples on the right side of Figs. 1 and 2 illustrate that, on average, attitudes toward immigration have become more positive in non-Eastern European countries in recent years, with 2018 recording the most positive attitudes toward immigration in both aspects. They also indicate a momentary drop in both attitudes in 2010 that can be associated with the Eurozone crisis. These findings are consistent with the entire sample, revealing that the Eurozone crisis was briefly associated with a decline in both immigration attitudes, while the refugee crisis had no negative association, with cultural attitudes becoming more positive since 2016.

There are stark differences, however, in the Eastern European sub-sample. Economically, the countries appear to have become less intolerant of immigration in 2018, but their attitudes are still decidedly negative (Fig. 2). Culturally, there has been a substantial decline in average net attitudes among the Eastern European countries, starting in 2014 and continuing until 2018 (Fig. 1). The sparklines indicate that, among the three countries, Hungary and Slovenia set the negative tone for the sub-sample in both aspects (red). In contrast, Poland shows a positive attitude toward immigration in cultural terms (blue) and a mixed trend in economic terms. This could be related to the fact that Hungary and Slovenia served as entry portals for refugees coming to Europe during the refugee crisis, while Poland was not directly affected by the influx of migrants. However, the refugee crisis can be linked to more negative economic attitudes in all three Eastern European countries. The Eurozone crisis, on the other hand, is not associated with a decline in either aspect of attitudes toward immigration, as the average scores in the sub-sample show no negative change in the years in question (Figs. 1 and 2). These results contradict the entire sample and the non-Eastern European sub-sample findings.

Overall, our descriptive results suggest that economic hardships rather than cultural changes that might be related to an increasing number of asylum seekers are more likely to generate negative attitudes toward immigration. This is an interesting finding, as during the 2015 refugee crisis, many asylum seekers arriving in Europe were fleeing persecution in Syria, Afghanistan, and Iraq—countries of predominantly Muslim origin (Kingsley, 2015; Wike et al., 2016). Nevertheless, the ongoing debate about the perceived rejection of liberal norms and values by Muslim immigrants (Bowen, 2014), which could have exacerbated the perception of a cultural threat, was not associated with a more negative attitude toward immigration in the whole sample. However, complementing previous studies (Bell et al., 2021), we found large discrepancies between non-Eastern European and Eastern European countries in both aspects of attitudes toward immigration. There were differences in reported net attitudes as such and in the potential impact of the Eurozone and refugee crises.

### Results of the analysis

**Fig. 3:**
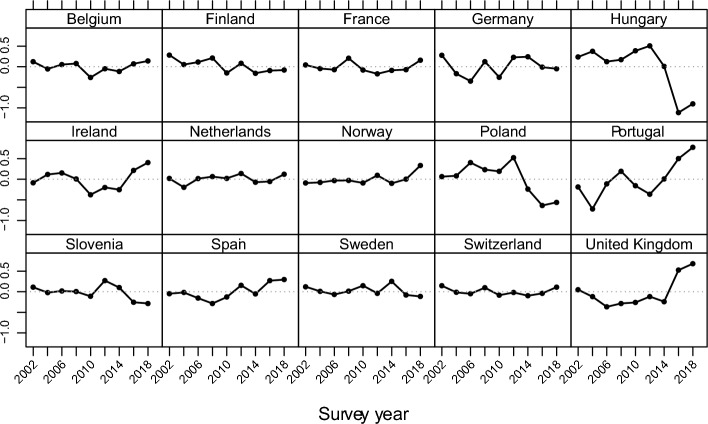
Period random effect estimates on cultural attitude from the hierarchical, three-level, cross-classified null model

**Fig. 4:**
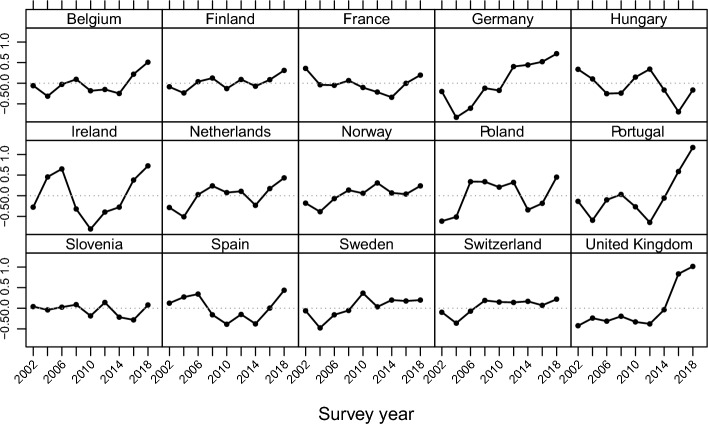
Period random effect estimates on economic attitude from the hierarchical, three-level, cross-classified null model

Our results from the hierarchical three-level cross-classified null model show that the smallest proportion of variance in cultural attitudes is explained by the period effect (country-period: 1.2%), followed by the cohort effect (country-cohort: 2.6%) (Model 0; Table 2), confirming the not too prominent role of the period already emphasised in our descriptive results. However, in the Eastern European countries, the period effect explains 4.2%, while in the non-Eastern European countries, it explains only 0.7% of the variance (Model 0; Tables 4 and 5), meaning that period effects manifest stronger in Eastern European countries than in their non-Eastern counterparts. This is different for economic attitudes. While the variance between country-cohort explains 1.1% and country-period 2.15% of the variance in economic attitudes in the pooled sample, the cohort effect only contributes to a small extent to explaining the variance in economic attitudes among the non-Eastern European countries (country-cohort: 0.9% and country-period: 2.2%) (Model 0; Tables 3 and 7). The contribution of the cohort and period effects is of a similar magnitude among the Eastern European countries (country-cohort: 2.5% and country-period: 2.0%) (Model 0; Tables 6). The random period effect illustrated in Fig. 3 only shows statistically significant negative changes in cultural attitudes after 2012 in Hungary and Poland and, to a smaller extent, in Slovenia. However, there have been significant changes over time in economic attitudes in most countries (Fig. 4). For example, in Germany, Ireland, Portugal, and the United Kingdom, economic attitudes became significantly more positive after 2010 and 2012.

**Table 2:** Hierarchical, three-level, crossed-classified random effects models of cultural attitudes toward immigration in 15 European countries

		Model 0	Model 1	Model 2a	Model 3a	Model 2b	Model 3b
	Intercept	5.767 (0.162) ***	5.962 (0.146) ***	6.148 (0.158) ***	5.960 (0.159) ***	5.61 (0.153) ***	5.652 (0.155) ***
Individual level	Education (ref: ISCED I)						
	ISCED II		0.347 (0.019) ***	0.347 (0.019) ***	0.524 (0.035) ***	0.347 (0.019) ***	0.333 (0.038) ***
	ISCED III		0.654 (0.018) ***	0.654 (0.018) ***	0.846 (0.032) ***	0.654 (0.018) ***	0.538 (0.038) ***
	ISCED IV		1.088 (0.022) ***	1.088 (0.022) ***	1.427 (0.040) ***	1.087 (0.022) ***	1.037 (0.045) ***
	ISCED V		1.656 (0.019) ***	1.656 (0.019) ***	1.862 (0.034) ***	1.656 (0.019) ***	1.665 (0.037) ***
Country-period level	Migrant inflow rate			− 0.244 (0.087) **	0.019 (0.093)		
	Unemployment rate					0.045 (0.009) ***	0.040 (0.010) ***
	ISCED II x migrant inflows				− 0.261 (0.042) ***		
	ISCED III x migrant inflows				− 0.289 (0.040) ***		
	ISCED IV x migrant inflows				− 0.453 (0.045) ***		
	ISCED V x migrant inflows				− 0.306 (0.041) ***		
	ISCED II x unemployment						0.001 (0.004)
	ISCED III x unemployment						0.014 (0.004) ***
	ISCED IV x unemployment						0.006 (0.005)
	ISCED V x unemployment						− 0.002 (0.004)
Random effects: var	Country (level 3)	0.337	0.282	0.272	0.270	0.238	0.237
	Country-period (level2)	0.083	0.101	0.095	0.095	0.085	0.085
	Country-cohort (level 2)	0.178	0.024	0.024	0.023	0.025	0.025
	Residual	6.323	5.529	5.529	5.527	5.529	5.529
	N	245,291	219,299	219,299	219,299	219,299	219,299

Higher scores indicate a more positive attitude; *p<0.05, **p<0.01, ***p<0.001; sampling weights are considered; model 0 represents the null model, model 1 presents the coefficients (S.E.) of education with ISCED I as reference category and individual characteristics as control variables; model 2 adds contextual variables migrant inflow rate (model 2a) and unemployment rate (model 2b); model 3 adds the interaction of education and contextual variables (migrant inflow rate in model 3a and unemployment rate in model 3b); the complete table with a list of all individual-level control variables can be found in Table A2 in the appendix

**Table 3:** Hierarchical, three-level, crossed-classified random effects models of economic attitudes toward immigration in 15 European countries

		Model 0	Model 1	Model 2a	Model 3a	Model 2b	Model 3b
	Intercept	5.038 (0.146) ***	4.529 (0.122) ***	4.446 (0.133) ***	4.333 (0.135) ***	4.592 (0.141) ***	4.682 (0.143) ***
Individual level	Education (ref: ISCED I)						
	ISCED II		0.388 (0.019) ***	0.388 (0.019) ***	0.507 (0.034) ***	0.388 (0.019) ***	0.317 (0.037) ***
	ISCED III		0.636 (0.018) ***	0.636 (0.018) ***	0.736 (0.031) ***	0.636 (0.018) ***	0.498 (0.036) ***
	ISCED IV		1.062 (0.021) ***	1.061 (0.021) ***	1.240 (0.039) ***	1.062 (0.021) ***	1.018 (0.044) ***
	ISCED V		1.619 (0.018) ***	1.619 (0.018) ***	1.763 (0.033) ***	1.619 (0.018) ***	1.484 (0.036) ***
Country-period level	Migrant inflow rate			0.109 (0.075)	0.267 (0.081) **		
	Unemployment rate					− 0.008 (0.008)	− 0.018 (0.009) *
	ISCED II x migrant inflows				− 0.174 (0.040) ***		
	ISCED III x migrant inflows				− 0.152 (0.038) ***		
	ISCED IV x migrant inflows				− 0.243 (0.043) ***		
	ISCED V x migrant inflows				− 0.210 (0.039) ***		
	ISCED II x unemployment						0.008 (0.003) *
	ISCED III x unemployment						0.016 (0.004) ***
	ISCED IV x unemployment						0.004 (0.004)
	ISCED V x unemployment						0.015 (0.003) ***
Random effects: var	Country (level 3)	0.287	0.192	0.186	0.185	0.203	0.203
	Country-period (level2)	0.139	0.070	0.069	0.069	0.069	0.069
	Country-cohort (level 2)	0.073	0.018	0.018	0.018	0.018	0.018
	Residual	5.974	5.108	5.108	5.107	5.108	5.108
	N	244,946	218,672	218,672	218,672	218,672	218,672

Higher scores indicate a more positive attitude; *p<0.05, **p<0.01, ***p<0.001; sampling weights are considered; model 0 represents the null model, model 1 presents the coefficients (S.E.) of education with ISCED I as reference category and individual characteristics as control variables; model 2 adds contextual variables migrant inflow rate (model 2a) and unemployment rate (model 2b); model 3 adds the interaction of education and contextual variables (migrant inflow rate in model 3a and unemployment rate in model 3b); the complete table with a list of all individual-level control variables can be found in Table A3 in the appendix

When individual characteristics are added (Model 1; Tables 2 and 3), our results suggest that respondents with a higher level of education are significantly more sympathetic toward immigration in both aspects than those with a lower level of education. Thus, our results confirm education’s liberalising role in shaping cultural and economic immigration attitudes while controlling for individual characteristics such as demographics, political affiliation, and satisfaction with the economy. Interestingly, the magnitude of education’s liberalising role remains unchanged when migrant inflow rates are added (Model 2a; Tables 2 and 3). However, higher migrant inflow rates are statistically negatively significant for cultural attitudes (Model 2a, Table 2), whereas they have no significant effect on economic attitudes (Model 2a, Table 3). In addition, higher migrant inflow rates attenuate education’s liberalising role in shaping cultural attitudes, as indicated by a significant negative interaction between education and migrant inflow rates (Model 3a, Table 2). The influx of migrants has no significant influence on economic attitudes. However, the interaction between education and migrant inflow rates is significant, with higher migrant inflow rates slightly reducing education’s liberalising role in economic attitudes (Model 3a, Table 3). In addition, the attenuation of education’s liberalising role ensuing from higher migrant inflow rates is greater for the lower educated than for the higher educated.

Economic hardship, proxied by the unemployment rate, is significantly positive for cultural attitudes (Model 2b, Table 2) but has no significant interaction with education (Model 3b, Table 2). Interestingly, while the unemployment rate is insignificant for economic attitudes (Model 2b; Table 3), it supports education’s liberalising role in shaping economic attitudes (Model 3b, Table 3).

A look at the differences in the role of education and contextual factors for non-Eastern and Eastern European countries—countries with different migration histories—reveals that for cultural attitudes, migrant inflow rates are negatively significant in non-Eastern European countries while insignificant in Eastern European countries (Model 2a, Tables 4 and 5). In non-Eastern European countries, higher migrant inflow rates also reduce education’s liberalising role (Model 2b, Table 5). This is different for the economic hardship hypothesis. Higher unemployment rates are significantly positive for cultural attitudes in both non-Eastern and Eastern European countries (Model 3a, Tables 4 and 5), while the interaction is insignificant (Model 3b).

**Table 4:** Hierarchical, three-level, crossed-classified random effects models of cultural attitudes toward immigration in 3 Eastern European countries

		Model 0	Model 1	Model 2a	Model 3a	Model 2b	Model 3b
	Intercept	5.267 (0.392) **	5.476 (0.409) ***	5.777 (0.415) ***	5.965 (0.425) ***	4.835 (0.382) ***	4.716 (0.43) ***
Individual level	Education (ref: ISCED I)						
	ISCED II		− 0.048 (0.097)	− 0.048 (0.097)	− 0.24 (0.138)	− 0.048 (0.097)	0.19 (0.233)
	ISCED III		0.399 (0.098) ***	0.4 (0.098) ***	0.251 (0.138)	0.398 (0.098) ***	0.491 (0.231) *
	ISCED IV		0.706 (0.11) ***	0.706 (0.11) ***	0.497 (0.154) **	0.704 (0.11) ***	0.583 (0.256) *
	ISCED V		1.016 (0.101) ***	1.016 (0.101) ***	0.725 (0.143) ***	1.015 (0.101) ***	1.169 (0.237) ***
Country-period level	Migrant inflow rate			− 0.704 (0.432)	− 1.612 (0.629) *		
	Unemployment rate					0.076 (0.025) **	0.089 (0.033) **
	ISCED II x migrant inflows				0.948 (0.487)		
	ISCED III x migrant inflows				0.719 (0.473)		
	ISCED IV x migrant inflows				1.022 (0.527)		
	ISCED V x migrant inflows				1.443 (0.491) **		
	ISCED II x unemployment						− 0.024 (0.022)
	ISCED III x unemployment						− 0.009 (0.022)
	ISCED IV x unemployment						0.012 (0.024)
	ISCED V x unemployment						− 0.015 (0.023)
Random effects: var	Country (level 3)	0.405	0.413	0.33	0.327	0.225	0.222
	Country-period (level2)	0.213	0.257	0.243	0.245	0.198	0.197
	Country-cohort (level 2)	0.129	0.044	0.045	0.045	0.045	0.044
	Residual	4.754	4.535	4.535	4.533	4.535	4.534
	N	39,548	32,285	32,285	32,285	32,285	32,285

Higher scores indicate a more positive attitude; *p<0.05, **p<0.01, ***p<0.001; sampling weights are considered; model 0 represents the null model, model 1 presents the coefficients (S.E.) of education with ISCED I as reference category and individual characteristics as control variables; model 2 adds contextual variables migrant inflow rate (model 2a) and unemployment rate (model 2b); model 3 adds the interaction of education and contextual variables (migrant inflow rate in model 3a and unemployment rate in model 3b); the complete table with a list of all individual-level control variables can be found in Table A4 in the appendix

**Table 5:** Hierarchical, three-level, crossed-classified random effects models of cultural attitudes toward immigration in 12 non-Eastern European countries

		Model 0	Model 1	Model 2a	Model 3a	Model 2b	Model 3b
	Intercept	5.891 (0.166) ***	6.097 (0.158) ***	6.28 (0.165) ***	6.057 (0.166) ***	5.856 (0.165) ***	5.884 (0.166) ***
Individual level	Education (ref: ISCED I)						
	ISCED II		0.355 (0.02) ***	0.356 (0.02) ***	0.549 (0.039) ***	0.355 (0.02) ***	0.328 (0.04) ***
	ISCED III		0.642 (0.019) ***	0.642 (0.019) ***	0.815 (0.034) ***	0.642 (0.019) ***	0.561 (0.04) ***
	ISCED IV		1.098 (0.023) ***	1.099 (0.023) ***	1.497 (0.043) ***	1.097 (0.023) ***	1.073 (0.047) ***
	ISCED V		1.69 (0.02) ***	1.691 (0.02) ***	2.006 (0.036) ***	1.69 (0.02) ***	1.688 (0.038) ***
Country-period level	Migrant inflow rate			− 0.217 (0.072) **	0.083 (0.079)		
	Unemployment rate					0.032 (0.009) ***	0.029 (0.009) **
	ISCED II x migrant inflows				− 0.285 (0.045) ***		
	ISCED III x migrant inflows				− 0.27 (0.041) ***		
	ISCED IV x migrant inflows				− 0.516 (0.047) ***		
	ISCED V x migrant inflows				− 0.44 (0.043) ***		
	ISCED II x unemployment						0.003 (0.004)
	ISCED III x unemployment						0.01 (0.004) *
	ISCED IV x unemployment						0.002 (0.005)
	ISCED V x unemployment						0 (0.004)
Random effects: var	Country (level 3)	0.276	0.269	0.253	0.25	0.237	0.236
	Country-period (level2)	0.053	0.061	0.055	0.055	0.054	0.054
	Country-cohort (level 2)	0.191	0.025	0.025	0.023	0.026	0.026
	Residual	6.625	5.67	5.67	5.665	5.67	5.669
	N	205,743	187,014	187,014	187,014	187,014	187,014

Higher scores indicate a more positive attitude; *p<0.05, **p<0.01, ***p<0.001; sampling weights are considered; model 0 represents the null model, model 1 presents the coefficients (S.E.) of education with ISCED I as reference category and individual characteristics as control variables; model 2 adds contextual variables migrant inflow rate (model 2a) and unemployment rate (model 2b); model 3 adds the interaction of education and contextual variables (migrant inflow rate in model 3a and unemployment rate in model 3b); the complete table with a list of all individual-level control variables can be found in Table A5 in the appendix

For economic attitudes, higher migrant inflow rates significantly increase the liberalising role of education in the Eastern European countries while reducing it in the non-Eastern countries (Model 3a, Tables 6 and 7). While economic hardships decrease favourable economic attitudes toward immigration among the lower educated, higher education appears to mitigate this effect in the non-Eastern European countries, although it does not completely override it (Model 3b, Table 7). This relationship, however, is insignificant in the Eastern European countries (Model 3b, Table 6).

**Table 6:** Hierarchical, three-level, crossed-classified random effects models of economic attitudes toward immigration in 3 Eastern European countries

		Model 0	Model 1	Model 2a	Model 3a	Model 2b	Model 3b
	Intercept	4.302 (0.400) **	4.403 (0.399) ***	4.425 (0.426) ***	4.723 (0.434) ***	4.428 (0.434) ***	4.228 (0.478) ***
Individual level	Education (ref: ISCED I)						
	ISCED II		− 0.139 (0.098)	− 0.139 (0.098)	− 0.394 (0.14) **	− 0.139 (0.098)	0.08 (0.232)
	ISCED III		0.22 (0.098) *	0.22 (0.098) *	− 0.059 (0.139)	0.22 (0.098) *	0.466 (0.23) *
	ISCED IV		0.49 (0.11) ***	0.49 (0.11) ***	0.133 (0.156)	0.49 (0.11) ***	0.537 (0.256) *
	ISCED V		0.839 (0.102) ***	0.839 (0.102) ***	0.425 (0.144) **	0.839 (0.102) ***	0.951 (0.237) ***
Country-period level	Migrant inflow rate			− 0.053 (0.329)	− 1.535 (0.577) **		
	Unemployment rate					− 0.003 (0.019)	0.018 (0.028)
	ISCED II x migrant inflows				1.236 (0.503) *		
	ISCED III x migrant inflows				1.371 (0.489) **		
	ISCED IV x migrant inflows				1.758 (0.544) **		
	ISCED V x migrant inflows				2.054 (0.507) ***		
	ISCED II x unemployment						− 0.023 (0.022)
	ISCED III x unemployment						− 0.025 (0.022)
	ISCED IV x unemployment						− 0.006 (0.024)
	ISCED V x unemployment						− 0.011 (0.022)
Random effects: var	Country (level 3)	0.428	0.412	0.421	0.414	0.421	0.424
	Country-period (level2)	0.112	0.106	0.11	0.111	0.111	0.11
	Country-cohort (level 2)	0.146	0.024	0.024	0.024	0.024	0.024
	Residual	5.042	4.695	4.695	4.692	4.695	4.695
	N	39,653	32,290	32,290	32,290	32,290	32,290

Higher scores indicate a more positive attitude; *p<0.05, **p<0.01, ***p<0.001; sampling weights are considered; model 0 represents the null model, model 1 presents the coefficients (S.E.) of education with ISCED I as reference category and individual characteristics as control variables; model 2 adds contextual variables migrant inflow rate (model 2a) and unemployment rate (model 2b); model 3 adds the interaction of education and contextual variables (migrant inflow rate in model 3a and unemployment rate in model 3b); the complete table with a list of all individual-level control variables can be found in Table A6 in the appendix

**Table 7:** Hierarchical, three-level, crossed-classified random effects models of economic attitudes toward immigration in 12 non-Eastern European countries

		Model 0	Model 1	Model 2a	Model 3a	Model 2b	Model 3b
	Intercept	5.221 (0.109) ***	4.634 (0.111) ***	4.541 (0.126) ***	4.387 (0.127) ***	4.732 (0.137) ***	4.815 (0.139) ***
Individual level	Education (ref: ISCED I)						
	ISCED II		0.397 (0.019) ***	0.397 (0.019) ***	0.527 (0.037) ***	0.397 (0.019) ***	0.324 (0.038) ***
	ISCED III		0.632 (0.018) ***	0.632 (0.018) ***	0.726 (0.033) ***	0.632 (0.018) ***	0.497 (0.038) ***
	ISCED IV		1.078 (0.022) ***	1.077 (0.022) ***	1.317 (0.041) ***	1.078 (0.022) ***	1.049 (0.045) ***
	ISCED V		1.657 (0.019) ***	1.657 (0.019) ***	1.924 (0.035)***	1.657 (0.019) ***	1.507 (0.037) ***
Country-period level	Migrant inflow rate			0.109 (0.072)	0.318 (0.078)***		
	Unemployment rate					− 0.013 (0.009)	− 0.022 (0.009) *
	ISCED II x migrant inflows				− 0.192 (0.043)***		
	ISCED III x migrant inflows				− 0.155 (0.039)***		
	ISCED IV x migrant inflows				− 0.317 (0.045)***		
	ISCED V x migrant inflows				− 0.362 (0.041)***		
	ISCED II x unemployment						0.008 (0.004) *
	ISCED III x unemployment						0.015 (0.004) ***
	ISCED IV x unemployment						0.002 (0.005)
	ISCED V x unemployment						0.017 (0.004) ***
Random effects: var	Country (level 3)	0.111	0.124	0.121	0.12	0.143	0.142
	Country-period (level2)	0.146	0.058	0.057	0.057	0.056	0.057
	Country-cohort (level 2)	0.058	0.01	0.01	0.01	0.01	0.01
	Residual	6.154	5.162	5.162	5.159	5.162	5.161
	N	205,293	186,382	186,382	186,382	186,382	186,382

Higher scores indicate a more positive attitude; *p<0.05, **p<0.01, ***p<0.001; sampling weights are considered; model 0 represents the null model, model 1 presents the coefficients (S.E.) of education with ISCED I as reference category and individual characteristics as control variables; model 2 adds contextual variables migrant inflow rate (model 2a) and unemployment rate (model 2b); model 3 adds the interaction of education and contextual variables (migrant inflow rate in model 3a and unemployment rate in model 3b); the complete table with a list of all individual-level control variables can be found in Table A7 in the appendix

In sum, our results show that while education plays some liberalising and empowering role in shaping both cultural and economic attitudes, the association of contextual factors and attitudes toward immigrants varies between the two attitudes and across regions.

## Conclusion

Previous research on attitudes toward immigration has repeatedly identified education as one of the most stable predictors, with higher levels of education being associated with more inclusive attitudes toward immigration. However, cross-national research on how contextual and regional factors determine attitudes toward immigration in Europe is scarce (Dinesen & Hjorth, 2020; Dražanová, 2022), especially considering the well-established link between education and immigration attitudes. This study aimed to help fill this gap by examining repeated cross-sectional data from 15 European countries over 16 years while considering socioeconomic changes, such as changes in unemployment rates and migrant inflow rates, as well as regional differences between Eastern European and non-Eastern European countries. Our main question was how these contextual and regional variations affect the role of education in shaping cultural and economic attitudes toward immigration.

Consistent with our expectations and previous research, we find supporting evidence for our first hypothesis—a robust positive and significant association between higher education and pro-immigration attitudes in both aspects between 2002 and 2018. The strength of this association, however, depends on contextual and regional factors. When examining the entire sample of 15 countries, our results reveal that higher migrant inflow rates attenuate the liberalising role of education in shaping cultural attitudes, lending support for the cultural threat explanation and refuting the first part of our second hypothesis. This finding challenges the generality of previous assumptions that the cognitive enhancement and broader thinking horizons associated with higher education make cultural attitudes toward immigration more liberal (Ceobanu & Escandel, 2008; Hyman & Wright, 1979). Interestingly, the difference in economic attitudes between those with higher and lower levels of education—education’s liberalising role—also decreases with a greater influx of immigrants. While we did not expect a significantly larger gap in economic attitudes between higher and lower educated in our second hypothesis, our finding suggests a clear decline in education’s liberalising role. This could be because a larger influx of immigrants can be perceived as a fiscal burden on the welfare system and taxpayers among the higher educated (Hainmueller & Hopkins, 2014).

Furthermore, greater economic hardship appears to decrease favourable economic attitudes toward immigration, while the liberalising role of education slightly increases with greater economic hardship. However, contrary to our expectations, there is no significant interaction between education and unemployment for cultural attitudes. Thus, we find supporting evidence for our third hypothesis with regard to economic attitudes but no evidence for cultural attitudes.

A look at the regional differences reveals an interesting dynamic. Consistent with previous research, we find that Eastern European countries have increasingly more negative attitudes toward immigration than their non-Eastern European counterparts (Bell et al., 2021). Specifically, our descriptive results show that of the three Eastern European countries in our sample, Slovenia and Hungary were the most anti-immigration in both aspects among the Eastern European countries and all 15 selected countries. This could be partially because these countries served as transit points for refugees coming to Europe during the 2015 immigration crisis and partly due to the official anti-refugee policy in Hungary (Simonovits, 2020), which led to an increased influx of refugees to Slovenia (BBC News, 2015).

Furthermore, our descriptive results suggest that non-Eastern European countries are more susceptible to an economic rather than a cultural threat perception. In Eastern European countries, net attitudes are negative in terms of both cultural and economic aspects, with a particularly high susceptibility to economic threats. However, contrary to our expectations and previous research on general immigration attitudes (Dražanová, 2022; Gorodzeisky & Semyonov, 2018; Jeannet & Dražanová, 2023), our findings indicate that higher unemployment rates are significantly positively associated with cultural attitudes in both regions but are significantly negative for economic attitudes only in non-Eastern European countries. Moreover, a larger influx of migrants reduces the liberalising role of education in shaping cultural attitudes in non-Eastern European countries but not in Eastern European countries, with a negative average net attitude. In addition, higher immigration rates have a significantly negative effect on the economic attitudes of lower-educated Eastern Europeans compared to higher-educated Eastern Europeans while reducing the liberalising role of education among non-Eastern Europeans. Finally, economic hardship increases the liberalising role of education in shaping economic attitudes in non-Eastern European countries but is not significant in Eastern European countries.

In sum, our results highlight regional differences in the role of contextual factors in shaping cultural and economic attitudes toward immigration. They corroborate previous research indicating that the relationships determining attitudes toward immigration in non-Eastern European countries do not necessarily apply to Eastern European countries (Dražanová, 2017). These differences may be attributed to the fact that Eastern European countries lived for decades under a highly restrictive socialist system with an explicitly internationalist ideology, which left little room for the development of national identities. This context urges more comparative research on the established relationship between education and attitudes toward immigration in the two regions.

Despite the merits of this study, we recognise its limitations. First, while we discuss the liberalising role of education in shaping cultural and economic attitudes toward immigration, we by no means imply causality, i.e., a direct effect, in this relationship. Thus, at best, we can report an association between higher education and positive or negative attitudes toward immigration in both aspects. Although recent studies engaging with causality in this relationship (Cavaille & Marshall, 2019; Finseraas et al., 2018; Simon, 2022; Velásquez & Eger, 2022; Weber, 2022) report somewhat conflicting results, they are an important endeavour to understand the direct mechanisms that influence individual attitudes toward immigration. We believe that further exploration of these mechanisms in different aspects of immigration attitudes in a larger number of countries will help to broaden our understanding of whether this relationship is indeed causal or circumstantial and how different contextual and regional factors influence the hypothesised causality.

Second, our selection of countries was determined by the availability of data, and three Eastern European countries are insufficient to draw overarching conclusions for the whole of Eastern Europe. However, the striking differences between the Eastern European and non-Eastern European countries in our sample justify a deeper scholarly interest in how the established relationship between education and immigration attitudes varies across what appear to be such disparate regions. There seems to be a consensus that higher education not only empowers people by providing them with the necessary skills to compete in the labour market but also helps them develop a more critical and liberal mind. This study, however, shows that this is not universal. We believe that future research engaging with this question, using different data sources and methodological techniques, will deepen our understanding of the role of regional differences.

## Supplementary Information

Additional file 1.

## Data Availability

The main data set analysed in the current study is available in the European Social Survey (2002–2018) repository, https://www.europeansocialsurvey.org/downloadwizard/. Additional sources of data are: FAOSTAT, available at http://www.fao.org/faostat/en/#data/OA; OECD, available at https://stats.oecd.org/Index.aspx?DataSetCode=MIG; SiStat, available at https://pxweb.stat.si/SiStatDb/pxweb/en/10_Dem_soc/10_Dem_soc__05_prebivalstvo__40_selitve__05_05N10_meddrzavne/05N1008S.px/; and the World Bank, available at https://databank.worldbank.org/source/world-development-indicators.

## Abbreviation

ISCED: International Standard Classification of Education
