# Supplementary material for: Revisiting the role of education in attitudes toward immigration in different contexts in Europe
Source: Genus. 2025 Jan 3;81(1):1. doi: 10.1186/s41118-024-00238-9 (PMC11698806; doi:10.1186/s41118-024-00238-9)
Supplement: Supplementary file 1 — Additional file 1. [file 41118_2024_238_MOESM1_ESM.pdf]

Appendix

Statistical overview of migrant inflow rate:

Table A1: Statistical overview of migrant inflow rate by country and year prior to survey year, country mean and total mean:

Country	2001	2003	2005	2007	2009	2011	2013	2015	2017	MEAN
**Belgium**	0.64	0.66	0.73	0.87	0.95	1.07	1.05	1.14	0.96	0.90
**Finland**	0.21	0.18	0.24	0.33	0.34	0.38	0.44	0.39	0.43	0.33
**France**	0.18	0.23	0.22	0.35	0.34	0.36	0.39	0.38	0.38	0.31
**Germany**	0.84	0.74	0.71	0.71	0.75	1.04	1.37	2.47	1.67	1.14
**Hungary**	0.20	0.19	0.25	0.23	0.26	0.23	0.22	0.26	0.38	0.25
**Ireland**	0.85	1.07	1.60	**2.78**	1.13	0.73	0.89	1.06	1.20	1.26
**Netherlands**	0.59	0.45	0.39	0.49	0.63	0.71	0.73	0.94	1.08	0.67
**Norway**	0.56	0.59	0.68	1.13	1.17	1.43	1.32	1.14	0.94	1.00
**Poland**	**0.06**	0.08	0.10	0.11	0.11	0.11	0.12	0.23	0.34	0.14
**Portugal**	1.46	0.30	0.27	0.31	0.58	0.43	0.32	0.37	0.60	0.51
**Slovenia**	0.39	0.47	0.75	1.51	1.19	0.88	0.76	0.96	1.33	0.92
**Spain**	0.95	1.01	1.55	2.03	0.78	0.71	0.53	0.62	0.97	1.02
**Sweden**	0.49	0.54	0.57	0.91	0.90	0.80	0.99	1.17	1.26	0.85
**Switzerland**	1.41	1.29	1.28	1.85	1.72	1.80	1.92	1.81	1.63	1.63
**United Kingdom**	0.44	0.55	0.67	0.74	0.68	0.71	0.63	0.73	0.78	0.66
**TOTAL**										0.77

Full tables of our hierarchical, three-level, cross-classified random effect models, including estimates for the control variables:

Table A2: Hierarchical, three-level, crossed-classified random effects models of cultural attitudes toward immigration in 15 European countries

		Model 0	Model 1	Model 2a	Model 3a	Model 2b	Model 3b
	Intercept	5.767 (0.162) ***	5.962 (0.146) ***	6.148 (0.158) ***	5.960 (0.159) ***	5.61 (0.153) ***	5.652 (0.155) ***
In	Education (ref: ISCED I)						
	ISCED II		0.347 (0.019) ***	0.347 (0.019) ***	0.524 (0.035) ***	0.347 (0.019) ***	0.333 (0.038) ***

		Model 0	Model 1	Model 2a	Model 3a	Model 2b	Model 3b
	ISCED III		0.654 (0.018) ***	0.654 (0.018) ***	0.846 (0.032) ***	0.654 (0.018) ***	0.538 (0.038) ***
	ISCED IV		1.088 (0.022) ***	1.088 (0.022) ***	1.427 (0.040) ***	1.087 (0.022) ***	1.037 (0.045) ***
	ISCED V		1.656 (0.019) ***	1.656 (0.019) ***	1.862 (0.034) ***	1.656 (0.019) ***	1.665 (0.037) ***
	Age group (ref: AG 14-19)						
	AG 20-29		-0.461 (0.025) ***	-0.461 (0.025) ***	-0.450 (0.025) ***	-0.461 (0.025) ***	-0.460 (0.025) ***
	AG 30-39		-0.569 (0.028) ***	-0.568 (0.028) ***	-0.556 (0.028) ***	-0.569 (0.028) ***	-0.569 (0.028) ***
	AG 40-49		-0.578 (0.031) ***	-0.578 (0.031) ***	-0.565 (0.031) ***	-0.579 (0.031) ***	-0.580 (0.031) ***
	AG 50-59		-0.621 (0.034) ***	-0.619 (0.034) ***	-0.605 (0.034) ***	-0.621 (0.034) ***	-0.621 (0.034) ***
	AG 60-69		-0.757 (0.037) ***	-0.756 (0.037) ***	-0.741 (0.037) ***	-0.758 (0.037) ***	-0.757 (0.037) ***
	AG 70-79		-0.837 (0.041) ***	-0.835 (0.041) ***	-0.820 (0.041) ***	-0.839 (0.041) ***	-0.836 (0.041) ***
	AG 80+		-0.834 (0.049) ***	-0.832 (0.049) ***	-0.815 (0.048) ***	-0.836 (0.049) ***	-0.833 (0.049) ***
	Non-native		0.567 (0.017) ***	0.568 (0.017) ***	0.567 (0.017) ***	0.567 (0.017) ***	0.565 (0.017) ***
	Rural residence		-0.255 (0.011) ***	-0.254 (0.011) ***	-0.253 (0.011) ***	-0.255 (0.011) ***	-0.254 (0.011) ***
	Female		0.066 (0.01) ***	0.066 (0.01) ***	0.065 (0.010) ***	0.066 (0.01) ***	0.067 (0.010) ***
	Left-right political affiliation		-0.212 (0.002) ***	-0.212 (0.002) ***	-0.212 (0.002) ***	-0.212 (0.002) ***	-0.212 (0.002) ***
	Satisfaction with economy		0.186 (0.002) ***	0.186 (0.002) ***	0.187 (0.002) ***	0.187 (0.002) ***	0.187 (0.002) ***
**Country-period level**	Migrant inflow rate			-0.244 (0.087) **	0.019 (0.093)		
	Unemployment rate					0.045 (0.009) ***	0.040 (0.010) ***
	ISCED II x migrant inflows				-0.261 (0.042) ***		
	ISCED III x migrant inflows				-0.289 (0.040) ***		
	ISCED IV x migrant inflows				-0.453 (0.045) ***		
	ISCED V x migrant inflows				-0.306 (0.041) ***		
	ISCED II x unemployment						0.001 (0.004)
	ISCED III x unemployment						0.014 (0.004) ***
	ISCED IV x unemployment						0.006 (0.005)
	ISCED V x unemployment						-0.002 (0.004)
**Random effects: var.**	Country (level 3)	0.337	0.282	0.272	0.270	0.238	0.237
	Country-period (level2)	0.083	0.101	0.095	0.095	0.085	0.085
	Country-cohort (level 2)	0.178	0.024	0.024	0.023	0.025	0.025
	Residual	6.323	5.529	5.529	5.527	5.529	5.529
	**N**	245,291	219,299	219,299	219,299	219,299	219,299

Notes: higher scores indicate a more positive attitude; *p<0.05, **p<0.01, ***p<0.001; sampling weights are considered; model 0 represents the null model, model 1 presents the coefficients (S.E.) of education with ISCED I as reference category and individual characteristics as control variables; model 2 adds contextual variables migrant inflow rate (model 2a) and unemployment rate (model

2b); model [3] adds the interaction of education and contextual variables (migrant inflow rate in model 3a and unemployment rate in model 3b).

Table A3: Hierarchical, three-level, crossed-classified random effects models of economic attitudes toward immigration in 15 European countries

		**Model 0**	**Model 1**	**Model 2a**	**Model 3a**	**Model 2b**	**Model 3b**
	Intercept	5.038 (0.146) ***	4.529 (0.122) ***	4.446 (0.133) ***	4.333 (0.135) ***	4.592 (0.141) ***	4.682 (0.143) ***
**Individual level**	Education (ref: ISCED I)						
	ISCED II		0.388 (0.019) ***	0.388 (0.019) ***	0.507 (0.034) ***	0.388 (0.019) ***	0.317 (0.037) ***
	ISCED III		0.636 (0.018) ***	0.636 (0.018) ***	0.736 (0.031) ***	0.636 (0.018) ***	0.498 (0.036) ***
	ISCED IV		1.062 (0.021) ***	1.061 (0.021) ***	1.240 (0.039) ***	1.062 (0.021) ***	1.018 (0.044) ***
	ISCED V		1.619 (0.018) ***	1.619 (0.018) ***	1.763 (0.033) ***	1.619 (0.018) ***	1.484 (0.036) ***
	Age group (ref: AG 14-19)						
	AG 20-29		-0.383 (0.024) ***	-0.383 (0.024) ***	-0.378 (0.024) ***	-0.383 (0.024) ***	-0.381 (0.024) ***
	AG 30-39		-0.443 (0.027) ***	-0.443 (0.027) ***	-0.437 (0.027) ***	-0.443 (0.027) ***	-0.441 (0.027) ***
	AG 40-49		-0.382 (0.03) ***	-0.382 (0.03) ***	-0.375 (0.030) ***	-0.382 (0.03) ***	-0.381 (0.030) ***
	AG 50-59		-0.364 (0.032) ***	-0.365 (0.032) ***	-0.357 (0.032) ***	-0.364 (0.032) ***	-0.362 (0.032) ***
	AG 60-69		-0.383 (0.035) ***	-0.384 (0.035) ***	-0.376 (0.035) ***	-0.383 (0.035) ***	-0.380 (0.035) ***
	AG 70-79		-0.399 (0.038) ***	-0.4 (0.038) ***	-0.392 (0.038) ***	-0.399 (0.038) ***	-0.394 (0.038) ***
	AG 80+		-0.341 (0.046) ***	-0.342 (0.046) ***	-0.332 (0.046) ***	-0.34 (0.046) ***	-0.334 (0.046) ***
	Non-native		0.656 (0.016) ***	0.656 (0.016) ***	0.656 (0.016) ***	0.656 (0.016) ***	0.655 (0.016) ***
	Rural residence		-0.221 (0.01) ***	-0.222 (0.01) ***	-0.221 (0.010) ***	-0.221 (0.01) ***	-0.221 (0.010) ***
	Female		-0.262 (0.009) ***	-0.262 (0.009) ***	-0.263 (0.009) ***	-0.262 (0.009) ***	-0.262 (0.009) ***
	Left-right political affiliation		-0.148 (0.002) ***	-0.148 (0.002) ***	-0.148 (0.002) ***	-0.148 (0.002) ***	-0.148 (0.002) ***
	Satisfaction with economy		0.244 (0.002) ***	0.244 (0.002) ***	0.244 (0.002) ***	0.244 (0.002) ***	0.244 (0.002) ***
**Country-period level**	Migrant inflow rate			0.109 (0.075)	0.267 (0.081) **		
	Unemployment rate					-0.008 (0.008)	-0.018 (0.009) *
	ISCED II x migrant inflows				-0.174 (0.040) ***		
	ISCED III x migrant inflows				-0.152 (0.038) ***		
	ISCED IV x migrant inflows				-0.243 (0.043) ***		
	ISCED V x migrant inflows				-0.210 (0.039) ***		
	ISCED II x unemployment						0.008 (0.003) *
	ISCED III x unemployment						0.016 (0.004) ***
	ISCED IV x unemployment						0.004 (0.004)
	ISCED V x unemployment						0.015 (0.003) ***
**Random effe**	Country (level 3)	0.287	0.192	0.186	0.185	0.203	0.203
	Country-period (level2)	0.139	0.070	0.069	0.069	0.069	0.069

		Model 0	Model 1	Model 2a	Model 3a	Model 2b	Model 3b
	Country-cohort (level 2)	0.073	0.018	0.018	0.018	0.018	0.018
	Residual	5.974	5.108	5.108	5.107	5.108	5.108
	**N**	244,946	218,672	218,672	218,672	218,672	218,672

Notes: higher scores indicate a more positive attitude; *p<0.05, **p<0.01, ***p<0.001; sampling weights are considered; model 0 represents the null model, model 1 presents the coefficients (S.E.) of education with ISCED I as reference category and individual characteristics as control variables; model 2 adds contextual variables migrant inflow rate (model 2a) and unemployment rate (model 2b); model [3] adds the interaction of education and contextual variables (migrant inflow rate in model 3a and unemployment rate in model 3b).

Table A4: Hierarchical, three-level, crossed-classified random effects models of cultural attitudes toward immigration in 3 Eastern European countries

		Model 0	Model 1	Model 2a	Model 3a	Model 2b	Model 3b
	Intercept	5.267 (0.392) **	5.476 (0.409) ***	5.777 (0.415) ***	5.965 (0.425) ***	4.835 (0.382) ***	4.716 (0.43) ***
**Individual level**	Education (ref: ISCED I)						
	ISCED II		-0.048 (0.097)	-0.048 (0.097)	-0.24 (0.138)	-0.048 (0.097)	0.19 (0.233)
	ISCED III		0.399 (0.098) ***	0.4 (0.098) ***	0.251 (0.138)	0.398 (0.098) ***	0.491 (0.231) *
	ISCED IV		0.706 (0.11) ***	0.706 (0.11) ***	0.497 (0.154) **	0.704 (0.11) ***	0.583 (0.256) *
	ISCED V		1.016 (0.101) ***	1.016 (0.101) ***	0.725 (0.143) ***	1.015 (0.101) ***	1.169 (0.237) ***
	Age group (ref: AG 14-19)						
	AG 20-29		-0.494 (0.061) ***	-0.494 (0.061) ***	-0.492 (0.061) ***	-0.492 (0.061) ***	-0.506 (0.062) ***
	AG 30-39		-0.513 (0.071) ***	-0.512 (0.071) ***	-0.516 (0.071) ***	-0.51 (0.071) ***	-0.518 (0.072) ***
	AG 40-49		-0.474 (0.08) ***	-0.472 (0.081) ***	-0.479 (0.081) ***	-0.47 (0.081) ***	-0.475 (0.081) ***
	AG 50-59		-0.496 (0.088) ***	-0.493 (0.088) ***	-0.496 (0.088) ***	-0.49 (0.088) ***	-0.49 (0.088) ***
	AG 60-69		-0.607 (0.098) ***	-0.603 (0.098) ***	-0.603 (0.098) ***	-0.6 (0.098) ***	-0.598 (0.098) ***
	AG 70-79		-0.621 (0.11) ***	-0.617 (0.11) ***	-0.613 (0.11) ***	-0.613 (0.11) ***	-0.615 (0.11) ***
	AG 80+		-0.536 (0.138) ***	-0.53 (0.138) ***	-0.528 (0.138) ***	-0.527 (0.138) ***	-0.535 (0.138) ***
	Non-native		0.727 (0.104) ***	0.727 (0.104) ***	0.732 (0.104) ***	0.726 (0.104) ***	0.729 (0.104) ***
	Rural residence		-0.2 (0.029) ***	-0.2 (0.029) ***	-0.201 (0.029) ***	-0.2 (0.029) ***	-0.2 (0.029) ***
	Female		-0.025 (0.025)	-0.025 (0.025)	-0.026 (0.025)	-0.025 (0.025)	-0.025 (0.025)
	Left-right political affiliation		-0.061 (0.005) ***	-0.061 (0.005) ***	-0.06 (0.005) ***	-0.061 (0.005) ***	-0.061 (0.005) ***
	Satisfaction with economy		0.113 (0.006) ***	0.113 (0.006) ***	0.113 (0.006) ***	0.113 (0.006) ***	0.114 (0.006) ***
**Country-period**	Migrant inflow rate			-0.704 (0.432)	-1.612 (0.629) *		
	Unemployment rate					0.076 (0.025) **	0.089 (0.033) **
	ISCED II x migrant inflows				0.948 (0.487)		

		Model 0	Model 1	Model 2a	Model 3a	Model 2b	Model 3b
	ISCED III x migrant inflows				0.719 (0.473)		
	ISCED IV x migrant inflows				1.022 (0.527)		
	ISCED V x migrant inflows				1.443 (0.491) **		
	ISCED II x unemployment						-0.024 (0.022)
	ISCED III x unemployment						-0.009 (0.022)
	ISCED IV x unemployment						0.012 (0.024)
	ISCED V x unemployment						-0.015 (0.023)
**Random effects: var.**	Country (level 3)	0.405	0.413	0.33	0.327	0.225	0.222
	Country-period (level2)	0.213	0.257	0.243	0.245	0.198	0.197
	Country-cohort (level 2)	0.129	0.044	0.045	0.045	0.045	0.044
	Residual	4.754	4.535	4.535	4.533	4.535	4.534
	**N**	39,548	32,285	32,285	32,285	32,285	32,285

Notes: higher scores indicate a more positive attitude; *p<0.05, **p<0.01, ***p<0.001; sampling weights are considered; model 0 represents the null model, model 1 presents the coefficients (S.E.) of education with ISCED I as reference category and individual characteristics as control variables; model 2 adds contextual variables migrant inflow rate (model 2a) and unemployment rate (model 2b); model 3 adds the interaction of education and contextual variables (migrant inflow rate in model 3a and unemployment rate in model 3b).

Table A5: Hierarchical, three-level, crossed-classified random effects models of cultural attitudes toward immigration in 12 non-Eastern European countries

		Model 0	Model 1	Model 2a	Model 3a	Model 2b	Model 3b
	Intercept	5.891 (0.166) ***	6.097 (0.158) ***	6.28 (0.165) ***	5.856 (0.165) ***	6.057 (0.166) ***	5.884 (0.166) ***
**Individual level**	Education (ref: ISCED I)						
	ISCED II		0.355 (0.02) ***	0.356 (0.02) ***	0.355 (0.02) ***	0.549 (0.039) ***	0.328 (0.04) ***
	ISCED III		0.642 (0.019) ***	0.642 (0.019) ***	0.642 (0.019) ***	0.815 (0.034) ***	0.561 (0.04) ***
	ISCED IV		1.098 (0.023) ***	1.099 (0.023) ***	1.097 (0.023) ***	1.497 (0.043) ***	1.073 (0.047) ***
	ISCED V		1.69 (0.02) ***	1.691 (0.02) ***	1.69 (0.02) ***	2.006 (0.036) ***	1.688 (0.038) ***
	Age group (ref: AG 14-19)						
	AG 20-29		-0.455 (0.027) ***	-0.455 (0.027) ***	-0.455 (0.027) ***	-0.441 (0.027) ***	-0.454 (0.027) ***
	AG 30-39		-0.575 (0.031) ***	-0.574 (0.031) ***	-0.576 (0.031) ***	-0.556 (0.031) ***	-0.575 (0.031) ***
	AG 40-49		-0.592 (0.034) ***	-0.59 (0.034) ***	-0.594 (0.034) ***	-0.57 (0.034) ***	-0.594 (0.034) ***
	AG 50-59		-0.632 (0.037) ***	-0.629 (0.037) ***	-0.634 (0.037) ***	-0.606 (0.036) ***	-0.634 (0.037) ***
	AG 60-69		-0.763 (0.04) ***	-0.76 (0.04) ***	-0.766 (0.04) ***	-0.737 (0.04) ***	-0.766 (0.04) ***
	AG 70-79		-0.845 (0.044) ***	-0.84 (0.044) ***	-0.848 (0.044) ***	-0.818 (0.044) ***	-0.846 (0.045) ***
	AG 80+		-0.85 (0.053) ***	-0.845 (0.053) ***	-0.854 (0.053) ***	-0.822 (0.052) ***	-0.851 (0.053) ***

		Model 0	Model 1	Model 2a	Model 3a	Model 2b	Model 3b
	Non-native		0.553 (0.018) ***	0.553 (0.018) ***	0.553 (0.018) ***	0.552 (0.018) ***	0.551 (0.018) ***
	Rural residence		-0.261 (0.011) ***	-0.261 (0.011) ***	-0.261 (0.011) ***	-0.261 (0.011) ***	-0.261 (0.011) ***
	Female		0.081 (0.01) ***	0.081 (0.01) ***	0.081 (0.01) ***	0.079 (0.01) ***	0.082 (0.01) ***
	Left-right political affiliation		-0.24 (0.003) ***	-0.24 (0.003) ***	-0.24 (0.003) ***	-0.24 (0.003) ***	-0.24 (0.003) ***
	Satisfaction with economy		0.198 (0.003) ***	0.198 (0.003) ***	0.198 (0.003) ***	0.198 (0.003) ***	0.198 (0.003) ***
**Country-period level**	Migrant inflow rate			-0.217 (0.072) **	0.083 (0.079)		
	Unemployment rate					0.032 (0.009) ***	0.029 (0.009) **
	ISCED II x migrant inflows				-0.285 (0.045) ***		
	ISCED III x migrant inflows				-0.27 (0.041) ***		
	ISCED IV x migrant inflows				-0.516 (0.047) ***		
	ISCED V x migrant inflows				-0.44 (0.043) ***		
	ISCED II x unemployment						0.003 (0.004)
	ISCED III x unemployment						0.01 (0.004) *
	ISCED IV x unemployment						0.002 (0.005)
	ISCED V x unemployment						0 (0.004)
**Random effects: var.**	Country (level 3)	0.276	0.269	0.253	0.25	0.237	0.236
	Country-period (level2)	0.053	0.061	0.055	0.055	0.054	0.054
	Country-cohort (level 2)	0.191	0.025	0.025	0.023	0.026	0.026
	Residual	6.625	5.67	5.67	5.665	5.67	5.669
	**N**	205,743	187,014	187,014	187,014	187,014	187,014

Notes: higher scores indicate a more positive attitude; *p<0.05, **p<0.01, ***p<0.001; sampling weights are considered; model 0 represents the null model, model 1 presents the coefficients (S.E.) of education with ISCED I as reference category and individual characteristics as control variables; model 2 adds contextual variables migrant inflow rate (model 2a) and unemployment rate (model 2b); model 3 adds the interaction of education and contextual variables (migrant inflow rate in model 3a and unemployment rate in model 3b).

Table A6: Hierarchical, three-level, crossed-classified random effects models of economic attitudes toward immigration in 3 Eastern European countries

		Model 0	Model 1	Model 2a	Model 3a	Model 2b	Model 3b
	Intercept	4.302 (0.400) **	4.403 (0.399) ***	4.425 (0.426) ***	4.723 (0.434) ***	4.428 (0.434) ***	4.228 (0.478) ***
**Individual level**	Education (ref: ISCED I)						
	ISCED II		-0.139 (0.098)	-0.139 (0.098)	-0.394 (0.14) **	-0.139 (0.098)	0.08 (0.232)
	ISCED III		0.22 (0.098) *	0.22 (0.098) *	-0.059 (0.139)	0.22 (0.098) *	0.466 (0.23) *

		Model 0	Model 1	Model 2a	Model 3a	Model 2b	Model 3b
	ISCED IV		0.49 (0.11) ***	0.49 (0.11) ***	0.133 (0.156)	0.49 (0.11) ***	0.537 (0.256) *
	ISCED V		0.839 (0.102) ***	0.839 (0.102) ***	0.425 (0.144) **	0.839 (0.102) ***	0.951 (0.237) ***
	Age group (ref: AG 14-19)						
	AG 20-29		-0.586 (0.063) ***	-0.586 (0.063) ***	-0.578 (0.063) ***	-0.586 (0.063) ***	-0.593 (0.063) ***
	AG 30-39		-0.676 (0.072) ***	-0.675 (0.072) ***	-0.677 (0.072) ***	-0.676 (0.072) ***	-0.674 (0.072) ***
	AG 40-49		-0.603 (0.08) ***	-0.603 (0.08) ***	-0.607 (0.08) ***	-0.603 (0.08) ***	-0.598 (0.08) ***
	AG 50-59		-0.603 (0.086) ***	-0.602 (0.086) ***	-0.604 (0.086) ***	-0.603 (0.086) ***	-0.599 (0.086) ***
	AG 60-69		-0.742 (0.094) ***	-0.742 (0.094) ***	-0.738 (0.094) ***	-0.743 (0.094) ***	-0.739 (0.094) ***
	AG 70-79		-0.807 (0.104) ***	-0.806 (0.105) ***	-0.796 (0.105) ***	-0.807 (0.105) ***	-0.806 (0.105) ***
	AG 80+		-0.746 (0.131) ***	-0.744 (0.132) ***	-0.73 (0.132) ***	-0.746 (0.132) ***	-0.743 (0.132) ***
	Non-native		0.632 (0.106) ***	0.632 (0.106) ***	0.641 (0.106) ***	0.632 (0.106) ***	0.635 (0.106) ***
	Rural residence		-0.069 (0.029) *	-0.069 (0.029) *	-0.071 (0.029) *	-0.069 (0.029) *	-0.069 (0.029) *
	Female		-0.266 (0.025) ***	-0.266 (0.025) ***	-0.266 (0.025) ***	-0.266 (0.025) ***	-0.267 (0.025) ***
	Left-right political affiliation		-0.042 (0.005) ***	-0.042 (0.005) ***	-0.041 (0.005) ***	-0.042 (0.005) ***	-0.042 (0.005) ***
	Satisfaction with economy		0.202 (0.006) ***	0.202 (0.006) ***	0.202 (0.006) ***	0.202 (0.006) ***	0.202 (0.006) ***
**Country-period level**	Migrant inflow rate			-0.053 (0.329)	-1.535 (0.577) **		
	Unemployment rate					-0.003 (0.019)	0.018 (0.028)
	ISCED II x migrant inflows				1.236 (0.503) *		
	ISCED III x migrant inflows				1.371 (0.489) **		
	ISCED IV x migrant inflows				1.758 (0.544) **		
	ISCED V x migrant inflows				2.054 (0.507) ***		
	ISCED II x unemployment						-0.023 (0.022)
	ISCED III x unemployment						-0.025 (0.022)
	ISCED IV x unemployment						-0.006 (0.024)
	ISCED V x unemployment						-0.011 (0.022)
**Random effects: var.**	Country (level 3)	0.428	0.412	0.421	0.414	0.421	0.424
	Country-period (level2)	0.112	0.106	0.11	0.111	0.111	0.11
	Country-cohort (level 2)	0.146	0.024	0.024	0.024	0.024	0.024
	Residual	5.042	4.695	4.695	4.692	4.695	4.695
	**N**	39,653	32,290	32,290	32,290	32,290	32,290

Notes: higher scores indicate a more positive attitude; *p<0.05, **p<0.01, ***p<0.001; sampling weights are considered; model 0 represents the null model, model 1 presents the coefficients (S.E.) of education with ISCED I as reference category and individual characteristics as control variables; model 2 adds contextual variables migrant inflow rate (model 2a) and unemployment rate (model

2b); model 3 adds the interaction of education and contextual variables (migrant inflow rate in model 3a and unemployment rate in model 3b).

Table A7: Hierarchical, three-level, crossed-classified random effects models of economic attitudes toward immigration in 12 non-Eastern European countries

		**Model 0**	**Model 1**	**Model 2a**	**Model 2b3a**	**Model 2b**	**Model 3b**
	Intercept	5.221 (0.109) ***	4.634 (0.111) ***	4.541 (0.126) ***	4.387 (0.127) ***	4.732 (0.137) ***	4.815 (0.139) ***
**Individual level**	Education (ref: ISCED I)						
	ISCED II		0.397 (0.019) ***	0.397 (0.019) ***	0.527 (0.037) ***	0.397 (0.019) ***	0.324 (0.038) ***
	ISCED III		0.632 (0.018) ***	0.632 (0.018) ***	0.726 (0.033) ***	0.632 (0.018) ***	0.497 (0.038) ***
	ISCED IV		1.078 (0.022) ***	1.077 (0.022) ***	1.317 (0.041) ***	1.078 (0.022) ***	1.049 (0.045) ***
	ISCED V		1.657 (0.019) ***	1.657 (0.019) ***	1.924 (0.035) ***	1.657 (0.019) ***	1.507 (0.037) ***
	Age group (ref: AG 14-19)						
	AG 20-29		-0.35 (0.026) ***	-0.35 (0.026) ***	-0.342 (0.026) ***	-0.35 (0.026) ***	-0.346 (0.026) ***
	AG 30-39		-0.4 (0.029) ***	-0.4 (0.029) ***	-0.387 (0.029) ***	-0.399 (0.029) ***	-0.397 (0.029) ***
	AG 40-49		-0.335 (0.031) ***	-0.336 (0.031) ***	-0.322 (0.031) ***	-0.335 (0.031) ***	-0.333 (0.031) ***
	AG 50-59		-0.305 (0.033) ***	-0.306 (0.033) ***	-0.291 (0.033) ***	-0.305 (0.033) ***	-0.303 (0.033) ***
	AG 60-69		-0.297 (0.036) ***	-0.298 (0.036) ***	-0.283 (0.036) ***	-0.296 (0.036) ***	-0.293 (0.036) ***
	AG 70-79		-0.3 (0.039) ***	-0.302 (0.039) ***	-0.287 (0.039) ***	-0.299 (0.039) ***	-0.294 (0.039) ***
	AG 80+		-0.242 (0.046) ***	-0.243 (0.046) ***	-0.229 (0.046) ***	-0.24 (0.046) ***	-0.234 (0.046) ***
	Non-native		0.647 (0.017) ***	0.647 (0.017) ***	0.647 (0.017) ***	0.648 (0.017) ***	0.647 (0.017) ***
	Rural residence		-0.242 (0.011) ***	-0.242 (0.011) ***	-0.242 (0.011) ***	-0.242 (0.011) ***	-0.241 (0.011) ***
	Female		-0.258 (0.01) ***	-0.258 (0.01) ***	-0.26 (0.01) ***	-0.258 (0.01) ***	-0.259 (0.01) ***
	Left-right political affiliation		-0.168 (0.002) ***	-0.168 (0.002) ***	-0.168 (0.002) ***	-0.168 (0.002) ***	-0.168 (0.002) ***
	Satisfaction with economy		0.25 (0.002) ***	0.25 (0.002) ***	0.251 (0.002) ***	0.25 (0.002) ***	0.25 (0.002) ***
**Country-period level**	Migrant inflow rate			0.109 (0.072)	0.318 (0.078) ***		
	Unemployment rate					-0.013 (0.009)	-0.022 (0.009) *
	ISCED II x migrant inflows				-0.192 (0.043) ***		
	ISCED III x migrant inflows				-0.155 (0.039) ***		
	ISCED IV x migrant inflows				-0.317 (0.045) ***		
	ISCED V x migrant inflows				-0.362 (0.041) ***		
	ISCED II x unemployment						0.008 (0.004) *
	ISCED III x unemployment						0.015 (0.004) ***
	ISCED IV x unemployment						0.002 (0.005)
	ISCED V x unemployment						0.017 (0.004) ***
**Ran**	Country (level 3)	0.111	0.124	0.121	0.12	0.143	0.142

		Model 0	Model 1	Model 2a	Model 2b3a	Model 2b	Model 3b
	Country-period (level2)	0.146	0.058	0.057	0.057	0.056	0.057
	Country-cohort (level 2)	0.058	0.01	0.01	0.01	0.01	0.01
	Residual	6.154	5.162	5.162	5.159	5.162	5.161
	**N**	205,293	186,382	186,382	186,382	186,382	186,382

Notes: higher scores indicate a more positive attitude; *p<0.05, **p<0.01, ***p<0.001; sampling weights are considered; model 0 represents the null model, model 1 presents the coefficients (S.E.) of education with ISCED I as reference category and individual characteristics as control variables; model 2 adds contextual variables migrant inflow rate (model 2a) and unemployment rate (model 2b); model 3 adds the interaction of education and contextual variables (migrant inflow rate in model 3a and unemployment rate in model 3b).

Results for the control variables within our analysis:

Among the control variables, age appears to be negative and significant in both aspects across all samples and models. However, the association is not linear, confirming some previous studies (e.g., Coenders & Scheepers, 2003). This could be related to the generational effect emphasised in recent literature (e.g., Jeannet and Dražanová, 2019).

Gender displays an interesting variation as a predictor of attitudes toward immigration. While the gender differences were small but significantly positive for the cultural aspect (Table A2, Model 1), women showed a significantly less positive attitude toward immigration than men in the economic aspect (Table A3, Model 1). This finding raises the question of whether fear of competition in the labour market and unequal working conditions, such as unequal wages, could be responsible for these differences rather than a personal dislike of foreign groups.

Our results show that respondents living in small towns and rural areas have more negative attitudes toward immigration in both aspects than respondents living in large cities (Tables A2 and A3, Model 1). This finding is consistent with the premise of contact theory (Pettigrew, 1998), as large cities provide more opportunities to encounter immigrants and form attitudes based on personal experience rather than negative headlines about immigration in the media and political campaigns. However, it may also be due to the open-mindedness of city dwellers, who are more accepting of cultural diversity from the outset, and the greater employment opportunities that big cities tend to offer.

Non-natives have more favourable attitudes toward immigration than respondents born in the country where they live (natives) (Tables A2 and A3, Model 1). Right-wing political affiliation was significantly associated with more negative attitudes toward immigration in both aspects (Tables A2 and A3, Model 1), confirming previous studies (Harteveld et al., 2017). Further, higher satisfaction with the economy was significantly associated with more positive attitudes toward immigration. This corroborates some previous findings (Coenders et al., 2008; Kehrberg, 2007) and contrasts with others (Semyonov et al., 2006). Interestingly, this result is positive and significant for both economic and cultural aspects of attitudes toward immigration (Tables A2 and A3, Model 1).
